# The Problem of Shot Selection in Basketball

**DOI:** 10.1371/journal.pone.0030776

**Published:** 2012-01-25

**Authors:** Brian Skinner

**Affiliations:** Fine Theoretical Physics Institute, University of Minnesota, Minneapolis, Minnesota, United States of America; University of Maribor, Slovenia

## Abstract

In basketball, every time the offense produces a shot opportunity the player with the ball must decide whether the shot is worth taking. In this article, I explore the question of when a team should shoot and when they should pass up the shot by considering a simple theoretical model of the shot selection process, in which the quality of shot opportunities generated by the offense is assumed to fall randomly within a uniform distribution. Within this model I derive an answer to the question “how likely must the shot be to go in before the player should take it?” and I show that this lower cutoff for shot quality 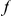 depends crucially on the number 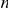 of shot opportunities remaining (say, before the shot clock expires), with larger 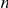 demanding that only higher-quality shots should be taken. The function 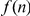 is also derived in the presence of a finite turnover rate and used to predict the shooting rate of an optimal-shooting team as a function of time. The theoretical prediction for the optimal shooting rate is compared to data from the National Basketball Association (NBA). The comparison highlights some limitations of the theoretical model, while also suggesting that NBA teams may be overly reluctant to shoot the ball early in the shot clock.

## Introduction

In the game of basketball, the purpose of an offensive set is to generate a high-quality shot opportunity. Thus, a successful play ends with some player from the offensive team being given the opportunity to take a reasonably high-percentage shot. At this final moment of the play, the player with the ball must make a decision: should that player take the shot, or should s/he retain possession of the ball and wait for the team to arrive at a higher-percentage opportunity later on in the possession?

The answer to this question depends crucially on three factors: (i) the (perceived) probability that the shot will go in, (ii) the distribution of shot quality that the offense is likely to generate in the future, and (iii) the number of shot opportunities that the offense will have before it is forced to surrender the ball to the opposing team (say, because of an expired shot clock). In this article I examine the simplest analytical model that accounts for all three of these factors and use it to derive a rule for shot selection in basketball.

Recent years have seen something of a revolution in analytical methods in sports, with advanced ideas from game theory, network theory, and statistical mechanics being used to highlight interesting phenomena associated with individual or cooperative performance [1]–[11]. The problem of shot selection in basketball has been a particularly popular subject of study [6], [7], [10]–[15]. Thus far, however, studies have generally focused on either the possible existence of “hot hand” phenomena [7], [10], [12]–[14] or on the choice between taking 2- and 3-point shots [6], [7], [15], and a general theoretical description of the shot-selection process has not been formulated. While the complex nature of decision-making in basketball makes such a description seem prohibitively difficult, it is nonetheless natural to describe the problem of shot selection in basketball as belonging to the class of “optimal stopping problems” (including, for example, the well-known “secretary problem”), which are often the domain of finance and, more broadly, decision theory and game theory [16].

A very recent work [11] has examined the shot selection problem using the perspective of “dynamic” and “allocative” efficiency criteria. The former criterion requires that every shot be taken only when its quality exceeds the expected point value of the remainder of the possession. The second criteria stipulates that, at optimum, all players on a team should have equal offensive efficiency. This allocative efficiency criterion is a source of some debate, as a recent paper [5] has suggested that the players' declining efficiency with increased usage implies an optimal shooting strategy that can violate the allocative efficiency criterion. Nonetheless, Ref. [11] demonstrates that players in the National Basketball Association (NBA) are excellent at shooting in a way that satisfies dynamic efficiency. That is, players' shooting *rates* seem to be consistent with their shooting *accuracy* when viewed from the requirement of maximizing dynamic efficiency. Still, there is no general theoretical model for addressing the question “when should a shot be taken and when should it be passed up?”.

Inspired by these recent discussions, in this article I construct a simple model of the “shoot or pass up the shot” decision and solve for the optimal probability of shooting at each shot opportunity. Within this model, each shot opportunity is characterized by its quality 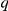, which is best defined as the expected number of points that will be scored if the shot as taken; in other words, 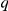 is the expected field goal percentage for a given shot multiplied by its potential point value (usually, 2 or 3). If all shots are taken to be worth 1 point, for example, then 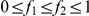. The possibility of offensive rebounds – whereby the team retains possession of the ball after a missed shot – is not considered explicitly in this article, but one can think that this possibility is lumped into the expected value of a given shot.

Given this definition, I make two important assumptions about the shot quality. The first assumption is that 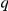 is a random variable, independent of all other shot opportunities, and is therefore described by some time-independent probability distribution. While this assumption remains somewhat controversial, thus far scoring trends have been shown to be predominately consistent with the assumption of statistical independence between successive shots [7], [10], [12], [17], with a weak “hot hand” effect having been seen only between successive free throw attempts [13], [14]. The second major assumption of the model, following Ref. [11], is that the probability distribution for 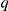 is a flat distribution: that is, at each shot opportunity 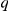 is chosen randomly between some minimum shot quality 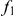 and some maximum 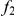. This assumption is somewhat arbitrary, and is chosen primarily for the sake of clarity and mathematical simplicity. In principle, however, one can generalize all results presented in this article to the case of a different statistical distribution for 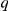. Some discussion about generalizations and limitations of the model is given at the end of this article in the Discussion section.

The primary concern of this article is calculating a rule for optimizing the shot selection process. That is, this article seeks to derive the optimal minimal value 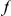 of the shot quality such that if players shoot if and only if the quality 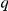 of the current shot satisfies 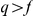, then their team's expected score per possession will be maximized. It should be noted that this goal of maximizing points per possession is appropriate for “risk neutral” situations, where teams are relatively evenly-matched and a significant amount of time remains in the game. The optimum strategy for end-game or “underdog” situations, where the team tries to maximize (or minimize) the probability of an unlikely upset, is considered in Ref. [15].

One can first note that this “lower cutoff” for shot quality 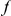 must depend on the number of plays 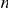 that are remaining in the possession. For example, imagine that a team is running their offense without a shot clock, so that they can reset their offense as many times as they want (imagine further, for the time being, that there is no chance of the team turning the ball over). In this case the team can afford to be extremely selective about which shots they take. That is, their expected score per possession is optimized if they hold on to the ball until an opportunity presents itself for a shot that is essentially certain to go in. On the other hand, if a team has time for only one or two shot opportunities in a possession, then there is a decent chance that the team will be forced into taking a relatively low-percentage shot.

So, intuitively, 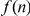 must increase monotonically with 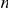. In the limit 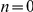 (when the current opportunity is the last chance for the team to shoot), we must have 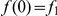; the team should be willing to take even the lowest quality shot. Conversely, in the limit 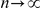 (and, again, in the absence of turnovers), 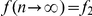; the team can afford to wait for the “perfect” shot. As I show below, the solution for 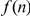 at all intermediate values of 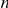 constitutes a non-trivial sequence that can only be defined recursively. I call this solution, 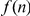, “the shooter's sequence”; it is the main result of the present article.

In the following Results section, I present the solution for 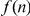, use it to derive a relation for the optimal shooting rate as a function of shot clock time, and then compare this theoretical result to data collected from NBA games. The Discussion section uses this comparison to suggest possible suboptimal behaviors among NBA players, and the limitations of the theoretical model are discussed along with some possible generalizations. Finally, the Methods section describes the collection and processing of the NBA data.

## Results

### 1 The shooter's sequence

In this subsection I calculate the optimal lower cutoff for shot quality, 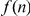, for a situation where there is enough time remaining for exactly 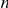 additional shot opportunities after the current one. I also calculate the expected number of points per possession, 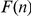, that results from following the optimal strategy defined by 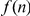. The effect of a finite probability of turning the ball over are considered in subsections 2–3.

To begin, we can first consider the case where the team is facing its last possible shot opportunity (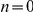). In this situation, the team should be willing to take the shot regardless of how poor it is, which implies 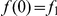. The expected number of points that results from this shot is the average of 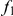 and 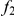 (the mean of the shot quality distribution):
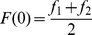
(1)


Now suppose that the team has enough time to reset their offense one time if they choose to pass up the shot; this is 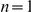. If the team decides to pass up the shot whenever its quality 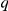 is below some value 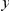, then their expected number of points in the possession is



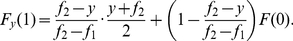
(2)In Eq. (2), the expression 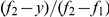 corresponds to the probability that the team will take the shot, so that the first term on the right hand side corresponds to the expected points per possession from shooting and the second term corresponds to the expected points per possession from passing up the shot. The optimal value of 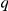, which by definition is equal to 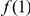, can be found by taking the derivative of 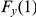 and equating it to zero:
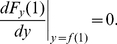
(3)


Combining Eqs. (2) and (3) gives 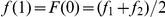. In other words, the team should shoot the ball whenever the shot opportunity has a higher quality 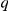 than the average of what they would get if they held the ball and waited for the next position. This is an intuitive and straightforward result. It can be extended to create a more general version of Eqs. (2) and (3). Namely,



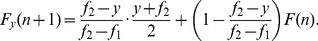
(4)and



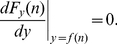
(5)Together, these two equations imply



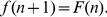
(6)This is the general statement that a team should shoot the ball only when the quality of the current opportunity is greater than the expected value of retaining the ball and getting 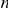 more shot opportunities. In this sense Eq. (6) is quite general, and it is independent of any assumptions about the distribution of available shot opportunities.

The conclusion of Eq. (6) allows one to rewrite Eq. (4) as a recursive sequence for 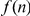:



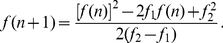
(7)Along with the initial value 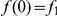, Eq. (7) completely defines “the shooter's sequence”. Surprisingly, considering the simplicity of the problem statement, this sequence 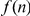 has no exact analytical solution. Its first few terms and its asymptotic limit are as follows:



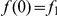


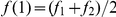


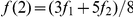


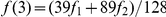


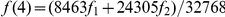


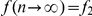



Note that in the limit where the team has infinite time, their shooting becomes maximally selective (only shots with “perfect” quality 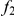 should be taken) and maximally efficient (every possession scores 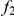 points).

Since Eq. (7) constitutes a recursive, quadratic map, it has no general solution [18]. Nonetheless, the expression for 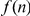 can be simplified somewhat by writing it in the form



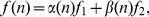
(8)where 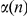 and 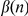 are separate recursive sequences defined by



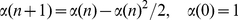
(9)and



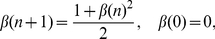
(10)respectively. While 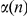 and 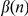 have no analytical solution, in the limit of large 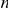 they have the asymptotic behavior 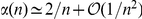 and 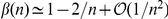.

### 2 Optimal shooting without a shot clock

In this subsection I consider situations in which there is no natural time limit to a possession, such as informal “pick-up” games. In this case, the number of shot opportunities that the team can generate is limited only by their propensity to turn the ball over – if the team attempts to continually reset the offense in search of a perfect shot they will eventually turn the ball over without taking any shots at all.

Thus, in these situations there is no natural definition of 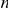, which implies that the solution for the optimal shot quality cutoff 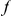 is a single number rather than a sequence. Its value depends on the upper and lower values of the distribution, 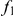 and 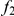, and on the probability 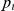 that the team will turn the ball over between two subsequent shot opportunities. To calculate 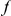, one can consider that the team's average number of points per possession, 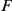, will be the same at the beginning of every offensive set, regardless of whether they have just chosen to pass up a shot. The team's optimal strategy is to take a shot whenever that shot's quality exceeds 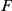; *i.e.*, 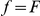 as in Eq. (6). This leads to the expression



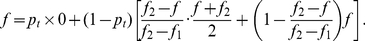
(11)In this equation, the term proportional to 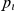 represents the expected points scored when the team turns the ball over (zero) and the term proportional to 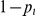 represents the expected points scored when the team does not turn the ball over. As in Eq. (4), the two terms inside the bracket represent the points scored when the shot is taken and when the shot is passed up.

Eq. (11) is a quadratic equation in 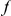, and can therefore be solved directly to give the optimal lower cutoff for shot quality in situations with no shot clock. This process gives



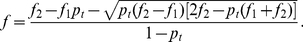
(12)For 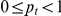 and 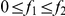, 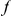 is real and positive. In the limit 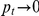, Eq. (12) gives 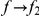 (perfect efficiency), as expected.

### 3 The shooter's sequence in the presence of turnovers

In this subsection I reconsider the problem of deriving the shooter's sequence while including the effect of a finite turnover probability 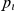. This constitutes a straightforward generalization of Eqs. (4) and (11). Namely,



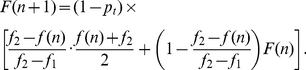
(13)Simplifying this expression and using Eq. (6) gives the recurrence relation



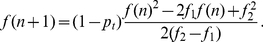
(14)Together with the condition 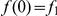, Eq. (14) completely defines the sequence 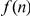.

Unfortunately, the sequence 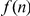 is unmanageable algebraically at all but very small 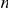. It can easily be evaluated numerically, however, if the values of 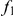, 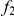, and 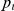 are known. The first few terms of 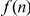 and its limiting expression are as follows:



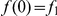


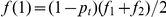


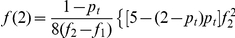


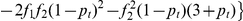


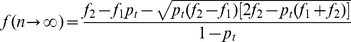



Notice that 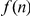 approaches the result of Eq. (12) in the limit where many shot opportunities remain (*i.e.* the very long shot clock limit).

Overall, the sequence 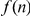 has two salient features: (i) it increases monotonically with 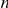 and ultimately approaches the “no shot clock” limit of Eq. (12), and (ii) it generally calls for the team to accept lower-quality shots than they would in the absence of turnovers, since the team must now factor in the possibility that future attempts will produce turnovers rather than random-quality shot opportunities.

### 4 Shooting rates of optimal shooters

The preceding subsections give the optimal shot quality cutoff as a function of the number of shots remaining. In this sense, the results presented above are useful for a team trying to answer the question “when should we take a shot?”. However, these results do not directly provide a way of answering the question “is the team shooting optimally?”. In other words, it is not immediately obvious how the shooter's sequence should manifest itself in shooting patterns during an actual game, where shot opportunities arise continuously in time.

When analyzing the shooting of a team based on collected (play-by-play) data, it is often instructive to look at the team's “shooting rate” 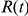. The shooting rate (also sometimes called the “hazard rate” [11]) is defined so that 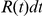 is the probability that a team with the ball at time 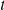 will shoot the ball during the infinitesimal interval of time 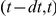. Here, 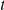 is defined as the time remaining on the shot clock, so that 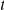 decreases as the possession goes on. In this subsection I calculate the optimum shooting rate 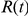 implied by the results for 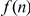. This calculation provides a means whereby one can evaluate how much a team's shooting pattern differs from the optimal one.

In order to calculate optimal shooting rate as a function of time, one should assume something about how frequently shot opportunities arise. In this subsection I make the simplest natural assumption, namely that shot opportunities arise randomly with some uniform rate 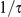. For example, 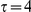 seconds would imply that on average a team gets six shot opportunities during a 24-second shot clock. The assumption of a uniform rate of shot opportunities is, in fact, unlikely to describe real data over the entire range of the shot clock, as discussed below in the Discussion section. Nonetheless, it allows one to derive analytically a number of important results. Possible generalizations from this assumption are discussed further at the end of this article in the Discussion section.

I also make the assumption that there is some uniform turnover rate 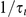. This assumption can easily be validated by examining turnover rates from NBA games, as discussed below in subsection 5.

Under this set of assumptions, one can immediately write down the probability 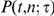 that at a given instant 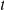 the team will have enough time for exactly 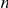 additional shot opportunities. Specifically, 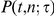 is given by the Poisson distribution:



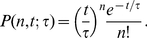
(15)


The probability 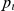 of a turnover between successive shot opportunities is given by



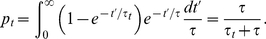
(16)The integrand in Eq. (16) contains the probability that there is at least one turnover during a time interval 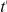 multiplied by the probability that there are no shot attempts during the time 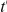 multiplied by the probability that a shot attempt arises during 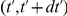, and this is integrated over all possible durations 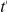 between subsequent shot attempts. While the upper integration limit in Eq. (16) should in principle be replaced by the total shot clock length 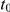, for 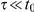 this limit can safely be set equal to 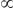.

In general, for a team deciding at a given time 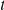 whether to shoot, the rate of shooting should depend on the proscribed optimal rate for when there are exactly 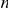 opportunities left, multiplied by the probability 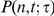 that there are in fact 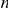 opportunities left, and summed over all possible 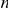. More specifically, consider that a team's optimal probability of taking a shot when there are exactly 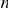 opportunities remaining is given by 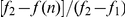, where 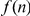 is the shooter's sequence defined by Eq. (14). The probability that the team should shoot during the interval 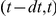 is therefore given by



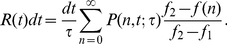
(17)Inserting Eq. (15) gives



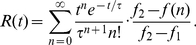
(18)Since the sequence 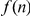 has no analytical solution, there is no general closed-form expression for 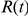.

The expected average efficiency (points/possession) of a team following the optimal strategy defined by 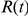 can be derived as follows. For a shot taken at time 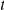, the optimal lower cutoff for shot quality, 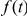, is given by 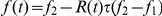. The corresponding average shot quality 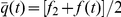 is given by



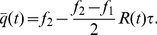
(19)


To find the expected number of points per possession, one needs to know the probability that a shot will be taken during a given time interval 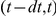. This quantity can be written as 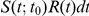, where 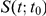 is the probability that the team still has the ball at time 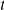 given that it gained possession at time 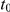 (the beginning of the shot clock).


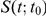
 can be derived by noting that the rate at which the current possession ends, 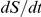, is given by the sum of the shooting rate and the turnover rate multiplied by the probability that the possession has not ended already:



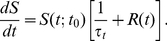
(20)Rearranging this equation and integrating gives



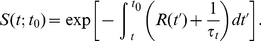
(21)Given this expression for 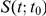 one can calculate the expected number of points scored during the possession, 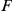, by integrating the average shot quality at time 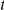 multiplied by the probability of a shot being taken during 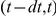 over all times 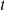. That is,



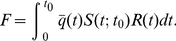
(22)While a closed-form analytical expression for 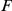 is not possible, Eq. (22) can easily be evaluated numerically.

**Figure 1:**
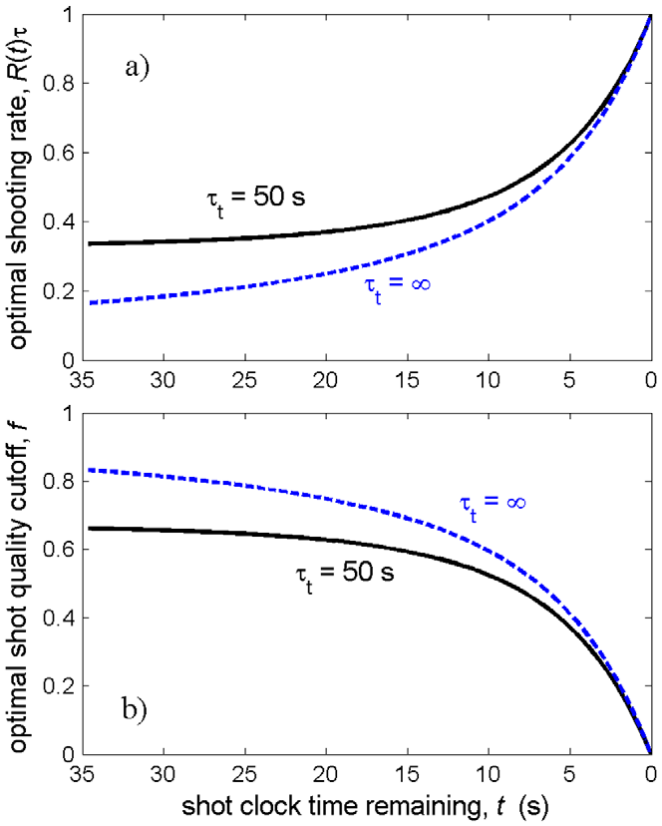
Optimal shooting rate and shot quality cutoff. a) Optimal shooting rate for a hypothetical team with 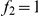, 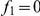, 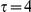 seconds, and 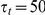 seconds, as given by Eq. (18). The shooting rate 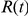 is plotted in the dimensionless form 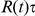, which can be thought of as the probability that a given shot that has arisen should be taken. The dashed line shows the hypothetical shooting rate for the team in the absence of turnovers. b) Optimal lower cutoff for shot quality, 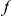, as a function of time for the same hypothetical team, both with and without a finite turnover rate.

As an example to illustrate optimal shooting behavior, consider a team that encounters shot opportunities with rate 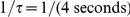 and turns the ball over with rate 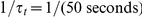. Using the sequence defined in Eq. (14), one can evaluate numerically the shooting rate implied by Eq. (18). This result is plotted as the black, solid line in Fig. 1a, using 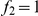 and 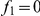. In Fig. 1a the optimal shooting rate is plotted as the dimensionless combination 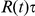, which can be thought of as the probability that a given shot should be taken if the opportunity arises at time 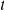 (as opposed to 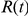, which is conditional on an opportunity presenting itself). For reference, I also plot the case where there are no turnovers, 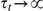. One can note that the finite turnover rate causes the optimal shooting rate to increase appreciably early in the shot clock. In other words, when there is a nonzero chance of turning the ball over the team cannot afford to be as selective with their shots.

The rule for optimal shooting can also be expressed in terms of the optimal lower cutoff for shot quality, 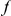, as a function of time. Since 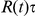 is the probability that a shot at 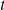 should be taken, 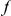 can be expressed simply as 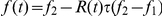. This optimal lower cutoff is plotted in Fig. 1b. A team that follows the optimal shooting strategy shown in Fig. 1 can be expected to score 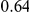 points per possession during games with a 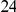-second shot clock [see Eq. (22)], a significant enhancement from the value 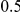 that might be naively expected by taking the average of the shot quality distribution.

In the limit of large time 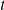 (or when there is no shot clock at all), as considered in subsection 2, the shooting rate 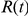 becomes independent of time and Eq. (18) has the following simple form:



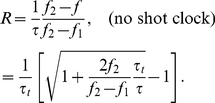
(23)Notice that when turnovers are very rare, 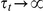, the shooting rate goes to zero, since the team can afford to be extremely selective about their shots.

Eq. (23) also implies an intriguingly weak dependence of the shooting rate on the average time 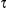 between shot opportunities. Imagine, for example, two teams, A and B, that both turn the ball over every 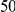 seconds of possession and both have shot distributions characterized by 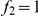, 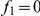. Suppose, however, that team A has much faster ball movement, so that team A arrives at a shot opportunity every 4 seconds while team B arrives at a shot opportunity only every 8 seconds. One might expect, then, that in the absence of a shot clock team A should have a shooting rate that is twice as large as that of team B. Eq. (23), however, suggests that this is not the case. Rather, team B should shoot on average every 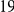 seconds and the twice-faster team A should shoot every 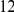 seconds. The net result of this optimal strategy, by Eqs. (12) and (16), is that team A scores 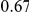 points per possession while team B scores 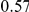 points per possession. In other words, team A's twice-faster playing style buys them not a twice-higher shooting rate, but rather an improved ability to be selective about which shots they take, and therefore an improved offensive efficiency.

### 5 Comparison to NBA data

Given the results of subsection 4, one can examine the in-game shooting statistics of basketball teams and evaluate the extent to which the teams' shooting patterns correspond to the ideal optimum strategy. In this subsection I examine data from NBA games and compare the measured shooting rates and shooting percentages of the league as a whole to the theoretical optimum rates derived above. The data are described in more detail in the Methods section.

**Figure 2:**
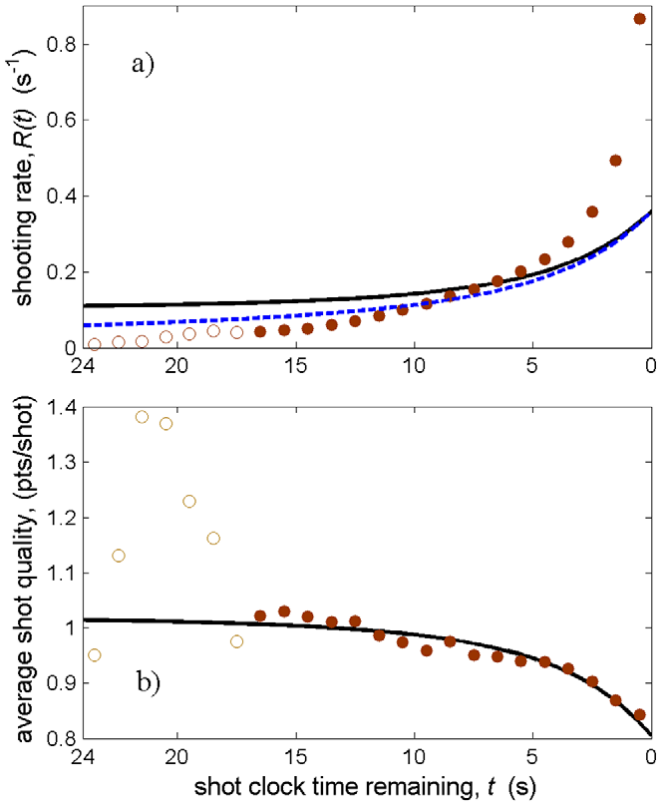
A comparison between the theoretical optimum shooting strategy and data from NBA games. a) The shooting rate as a function of shot clock time 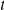. The solid black line corresponds to the parameters 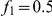, 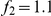, 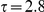 s, which are determined by a best fit to the shot quality data, using the NBA average turnover rate 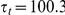 seconds. The dashed blue line corresponds to the same parameters except with the turnover rate 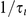 set to zero. b) The average quality (points per shot) of shots taken as a function of the shot clock time 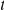. The solid line corresponds to the best fit curve to the filled symbols, from which the parameters for the solid black line in a) are determined.

The average shooting rate and shot quality (points scored per shot taken) for NBA players are plotted as the symbols in Fig. 2a and b, respectively, as a function of time. Open symbols correspond to shots taken during the first seven seconds of the shot clock, which generally correspond to “fast break” plays during which the offense is not well-described by the theoretical model developed in this article.

**Figure 3:**
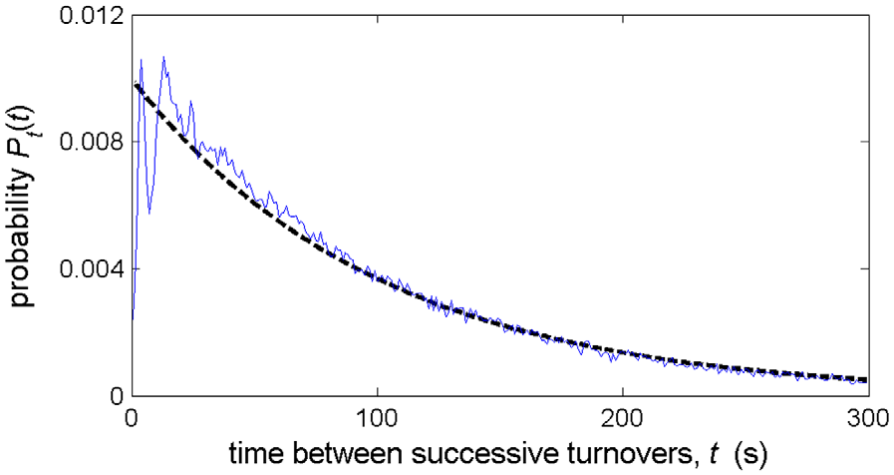
The probability distribution for the time between successive turnovers for NBA teams. The recorded distribution (thin blue line) is well described by the theoretical distribution 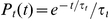 (thick black dashed line) corresponding to a uniform turnover rate 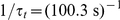.

In order to compare this data with the theoretical optimum behavior proscribed by the theories of the subsections 1 – 3, one should determine the values 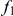, 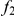, 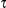, and 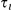 that best describe the average NBA offense. This last parameter, the average time between turnovers, can be extracted directly from the data: 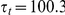 seconds, as illustrated in Fig. 3. The other parameters can be determined only implicitly, by fitting the observed shooting rates and percentages to the theoretical model.

For the curves shown in Fig. 2, the following approach is employed. First, the average shot quality for NBA teams is determined from the data as a function of time (Fig. 2b). Then, the theoretical average shot quality 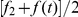 of an optimal-shooting team is fit to this data in order to determine the best-fit values of 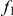, 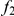, and 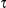, assuming optimal behavior. This procedure gives 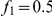, 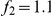, and 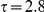 seconds. The corresponding fit line is shown as the solid curve in Fig. 2b. The shooting rate 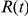 implied by these parameter values is then calculated and compared to the shooting rate measured from NBA games (Fig. 2a). In this way one can compare whether the measured shooting *rates* of NBA teams are consistent with their shooting *percentages*, within the assumptions of the theoretical model.

The result, as shown in Fig. 2a, is that NBA teams have a noticeably lower shooting rate during the early stages of the shot clock than is proscribed by the theoretical model. With 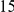 seconds remaining on the shot clock, for example, the average NBA team has a probability of only about 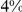 of shooting the ball during the next second, whereas the optimal strategy suggests that this probability should be as high as 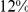. This observation is in qualitative agreement with the findings of Ref. [11], which concludes that under-shooting is far more common in the NBA than over-shooting. At small 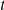, the large gap between the observed and theoretical shooting rates suggests a breakdown of the theoretical model, as discussed in the following section.

The difference between the actual and optimal shooting rates is also reflected in the average scoring efficiency 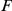. For NBA teams, the expected number of points per possession is 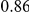, or 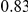 if one considers only possessions lasting past the first seven seconds of the shot clock. In contrast, the optimal shooting strategy shown by the solid lines in Fig. 2 produces 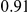 points/possession for a 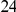-second shot clock and 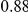 points/possession for a 17-second clock [see Eq. (22)], even though it corresponds to the same distribution of shot quality. This improvement of 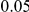 points/possession translates to roughly 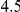 points per game. According to the established “Pythagorean” model of a team's winning percentage in the NBA [19], such an improvement could be expected to produce more than 10 additional wins for a team during an 82-game season.

## Discussion

If one operates under the assumption that the theoretical prediction derived in the previous section indeed provides a meaningful comparison with NBA data, then one natural way to interpret the discrepancy between the observed and the theoretically optimal shooting behavior of NBA teams is as a sign of overconfident behavior. That is, NBA players may be unwilling to settle for only moderately high-quality shot opportunities early in the shot clock, believing that even better opportunities will arise later. Part of the discrepancy can also be explained in terms of undervaluation of turnover rates. If the players believe, for example, that their team has essentially no chance of turning the ball over during the current possession, then they will be more likely to hold the ball and wait for a later opportunity. This effect is illustrated by the dashed blue line in Fig. 2a, which shows the optimal shooting rate for the hypothetical case 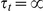 (the absence of turnovers). This line is in significantly better agreement with the observed shooting rates at large 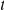, which suggests the possibility that when NBA teams make their shooting decisions early in the shot clock they do not account for the probability of future turnovers.

Of course, it is possible that much of the disagreement between the observed and theoretically optimum shooting rates can be attributed to an inaccuracy in the theory's assumption (in subsection 4 above) that shot opportunities arise randomly in time. The breakdown of this assumption can be seen in particular at small 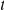 in Fig. 2a, where the shooting rate exceeds 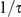, the supposed rate at which shot opportunities arise. This discrepancy can be seen as an indication that NBA teams often run their offense so as to produce more shot opportunities as the clock winds down, which results in shooting rates that are weighted more heavily toward later times. It is also likely that at very small time 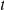 the theory's assumption of a uniform distribution of shot quality becomes invalid. Indeed, in these “buzzer-beating” situations the teams' shots are often forced, and their quality is likely not chosen from the same random distribution as for shots much earlier in the shot clock.

In this sense, the theoretical result of Eq. (18) cannot be considered a very exact description of the shooting rates of NBA teams. In order to improve the applicability of the model for real-game situations, one should account for the possibility of time dependence in the shot quality distribution (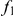 and 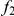) and the rate of shot opportunities (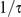). Such considerations are beyond the scope of the present work.

More generally, a major limitation of the model presented here is its reliance on somewhat arbitrary assumptions about the distribution of shot quality 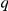 and about the rate at which these shot opportunities arise. This article has made the simplest set of assumptions – a uniform, time-independent distribution and a uniform rate – but real game situations are likely to be more complex. Unfortunately, the shot quality distribution and rate of generation of shot opportunities cannot be extracted in a straightforward way from existing data. Specifically, game data records only the outcome of shots that were *taken*, and not the quality of opportunities that were passed up. In this way there is no obvious way to gain information about which shot opportunities present themselves without making some assumptions about the players' decision making.

Nonetheless, it should be noted that the model presented here can easily be extended to accommodate different assumptions about the the shot quality distribution and the rate of shot opportunities. Generally speaking, if one assumes that the shot quality distribution is characterized by some probability density function 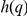, then the recurrence relation of Eq. (22) becomes



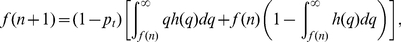
(24)so that the entire shooter's sequence can be calculated recursively. [Inserting the flat distribution 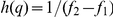 into Eq. (24) reproduces Eq. (14).] The corresponding optimal shooting rate as a function of time can also be calculated by replacing the Poisson distribution 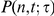 in Eq. (18) with some other distribution that is assumed to describe the rate at which shot opportunities arise.

Notwithstanding these complications, the model presented in this article nonetheless provides a useful first approach to describing theoretically the problem of shot selection in basketball, and it may be helpful in predicting how optimal strategy should adapt to changing features of the offense – *e.g.* an altered pace of play (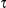) or an improving/declining team shooting ability (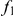 and 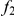) or a changing turnover rate (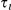). If nothing else, the theory developed in this article may pave the way for a more complex and accurate theoretical model in the future. In this way the problem of shot selection in basketball should be added to the interesting and growing literature on optimal stopping problems. More broadly, the question of optimal behavior in sports continues to provide an interesting, novel, and highly-applicable playground for mathematics and statistical mechanics.

## Methods

The data presented in Figs. 3 and 2 is based on recorded play-by-play data from 4,720 NBA games during the 2006–2007–2009–2010 seasons. These data are available publicly at http://www.basketballgeek.com/data/. From these data, shots taken and points scored are sorted for all possessions based on how much time remains on the shot clock at the time of the shot. Following Ref. [11], possessions that occur within the last 24 seconds of a given quarter or within the last six minutes of a game are eliminated from the data set, since these are less likely to correspond to risk-neutral situations. I also exclude from the data set all shots for which the shot clock time cannot be accurately inferred. These include shots that immediately follow an offensive rebound, defensive foul, or timeout.

The data presented in Figs. 2 and 3 correspond to the average behavior for the NBA as a whole. While a systematic breakdown of shooting rates by team is outside the scope of this article, I note briefly that the shooting rate 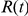 is essentially invariant between NBA teams to within statistical noise.
